# TIMP-2 gene rs4789936 polymorphism is associated with increased risk of breast cancer and poor prognosis in Southern Chinese women

**DOI:** 10.18632/aging.103789

**Published:** 2020-10-07

**Authors:** Gaoming Liu, Jiayou Luo

**Affiliations:** 1 Department of Maternal and Child Health, Xiangya School of Public Health, Central South University, Changsha 410078, Hunan Province, China; 2 Department of Women’s Cancer, Hunan Cancer Hospital, The Affiliated Cancer Hospital of Xiangya School of Medicine, Central South University, Changsha 410013, Hunan, China

**Keywords:** breast cancer, gene polymorphism, TIMP-2, survival analysis, prognosis

## Abstract

Tissue inhibitor of metalloproteinase-2 (TIMP-2) is an endogenous inhibitor of matrix metalloproteinase-2, and an important regulator of cancer growth and metastasis. Here, we evaluated the relationship between TIMP-2 gene rs4789936 polymorphism and breast cancer risk and prognosis in Southern Chinese women. Analysis of 480 breast cancer cases and 530 healthy controls revealed that compared to the CC genotype, the TT/CT genotypes increased the risk of breast cancer (CT: odds ratio (OR)=1.46, 95%CI: 1.11-1.92; TT: OR=2.57, 95%CI: 1.76-3.77). Stratified and cross-over analyses indicated that the increased risk of the rs4789936 polymorphism was associated with age, smoking, drinking, and the body mass index. The TT and CT genotypes were more common than the CC genotype in patients with lymph node metastases (TT: OR=2.54, 95%CI: 1.54-4.18; CT: OR=2.10, 95%CI: 1.40-3.15), advanced stage (TT: OR=1.91, 95%CI: 1.17-3.14; CT: OR=1.80, 95%CI: 1.20-2.70), or progesterone receptor positive cancer (TT: OR=2.02, 95%CI: 1.23-3.32). Multivariate cox regression indicated the CT and TT genotypes (HR=4.65, 95%CI: 1.26-17.18, P=0.021; HR=6.62, 95%CI: 1.51-29.11, P=0.012) were associated with poor prognosis in breast cancer patients. Thus, the TIMP-2 gene rs4789936 polymorphism is associated with elevated breast cancer risk and may be an independent prognosis factor in breast cancer patients.

## INTRODUCTION

Breast cancer is the most common cancer in women worldwide [1], as well as in China [2]. The incidence of breast cancer in China continues to rise rapidly at a rate of 3% to 4% per year, which is double the global average [3]. Even though the breast cancer treatment in China has greatly improved, the overall survival rates are still lower than in other countries [4]. The increasing incidence of breast cancer, the huge population base, and the lower survival prognosis, represent a severe challenge for the prevention and control of breast cancer in China. Therefore, identifying the environmental and genetic risk factors of breast cancer in China is very important to reduce the incidence of breast cancer.

Occurrence and development of breast cancer are affected by many genetic and environmental factors and their interactions [5]. Many potential environmental risk factors of breast cancer have been identified, including smoking [6], drinking [7], diet [8], and hormone exposure [9]. In addition, genetic factors play a very important role in the development and progression of breast cancer [10]. Previous studies have suggested that the increased risk of breast cancer is associated with breast cancer susceptibility gene 1 [11], leptin G-2548A gene [12], interleukin 4 (IL-4) [13], and insulin-like growth factor 1 [14].

Tissue inhibitor of metalloproteinase-2 (TIMP-2) is an endogenous inhibitor of matrix metalloproteinase-2 (MMP-2)[15], an important enzyme in the regulation of cancer cell proliferation and metastasis [16]. MMP-2 is over-expressed in cancer tissues, and its increased expression correlates with advanced cancer stage and poor prognosis. TIMP-2 forms a complex with MMP-2, thus regulating MMP-2 proteolytic activity. TIMP-2 mutations on chromosome 17q25 can affect its binding to MMP-2, resulting in cancer development [17]. Previous studies have indicated that TIMP-2 gene polymorphism is associated with gastric cancer [18], lung cancer [19], and prostate cancer [20]. In addition, TIMP-2 gene rs4789936/CT genotype is associated with an increased risk of colorectal cancer compared with CC genotype [21]. A recent study from Northern Chinese population has suggested that the mutation of TIMP-2 gene rs4789936 polymorphism is not associated with an increased risk of breast cancer [22]. However, the results remain controversial due to the different eating habits, environmental factors, and genetic mutation frequency. In this study, we evaluated the relationship between TIMP-2 gene rs4789936 polymorphism and the risk of breast cancer in Southern Chinese population. We also performed a follow-up study to determine the effect of rs4789936 polymorphism on breast cancer prognosis.

## RESULTS

### Characteristics of study population

The study included 480 breast cancer patients and 530 healthy controls; the main characteristics of the patient case group and the healthy control group are presented in Table 1. There were no significant differences in age (P=0.110), BMI (P=0.405), and drinking (P=0.283). Notably, the distribution of drinking population was significantly different between the case group and the control group (19.4% vs 13.6%, P=0.013). Among the case population, 52.1% of breast cancer patients had tumors with diameter ≥ 2cm, 54.2% of patients belonged to stage I/II, 42.7% of patients were ER positive, 48.5% were PR positive, and 67.5% were her-2 positive. Follow-up revealed that 33.8% of patients received radiation alone, and 66.3% received chemotherapy and radiotherapy.

**Table 1:** General characteristics of case group and control group.

**Parameters**	**Case group**	**Control group**	**χ^2^/t**	**P**
Age(y)	49.6±7.0	50.3±6.9	1.598	0.110
BMI (kg/m^2^)	23.6±3.0	23.7±3.2	0.832	0.405
Smoking (n, %)	93(19.4%)	72(13.6%)	6.178	0.013
Drinking (n, %)	115(24.0%0	112(21.1%)	1.1555	0.283
Tumor size, cm				
<2cm	230(47.9%)			
≥2cm	250(52.1%)			
Lymph node metastases				
Yes	231(48.1%)			
No	249(51.9%)			
Stage				
I/II	260(54.2%)			
III/IV	220(45.8%)			
ER				
Positive	205(42.7%)			
Negative	275(57.3%)			
PR				
Positive	233(48.5%)			
Negative	247(51.5%)			
Her-2				
Positive	324(67.5%)			
Negative	156(32.5%)			
Treatment				
Radiation	162(33.8%)			
Chemoradiotherapy	318(66.3%)			

ER, estrogen receptor; PR, progesterone receptor.

### TIMP-2 gene rs4789936 polymorphism and breast cancer risk

The genotype of control group did not exhibit a departure from Hardy-Weinberg equilibrium (P>0.05). The genotype distributions of case and control groups are presented in Table 2. Compared with CC genotype, the patients with CT/TT genotype had an elevated risk of breast cancer (CT: OR=1.46, 95%CI:1.11-1.92, P=0.007; TT: OR=2.57, 95%CI: 1.76-3.77, P<0.001). Significant differences were also found using dominant model (CT+TT: OR=1.71, 95%CI: 1.33-2.20, P<0.001), recessive model (TT vs CT+CC: OR=2.19, 95%CI: 1.52-3.15, P<0.001), and allele model (T vs C: OR=1.69, 95%CI: 1.40-2.04, P<0.001). After adjusting for age, smoking, drinking, and BMI, significant differences were still observed. Detailed results are presented in Table 2. Stratified analysis (Table 3) indicated a significantly increased breast cancer risk in the homozygote model (TT vs CC) that was associated with age, smoking, drinking, and BMI. The heterozygote model (CT vs CC) was significant among population of <60 years, non-smokers and non-drinkers. For BMI>24, the dominant model showed an increased risk of breast cancer.

**Table 2:** Genotype of frequencies of TIMP-2 gene rs4789936 in cases and controls.

**Modes**	**Genotype**	**Case (n, %)**	**Control (n, %)**	**OR (95%CI)**	***P***	OR (95%CI)*	P*
rs4789936							
Co-dominant	CC	204(42.5%)	296(55.8%)	1.00			
Heterozygote	CT	182(37.9%)	181(34.2%)	1.46(1.11-1.92)	0.007	1.45(1.10-1.91)	0.007
Homozygote	TT	94(19.6%)	53(10.0%)	2.57(1.76-3.77)	<0.001	2.41(1.63-3.55)	<0.001
Dominant	CC	204(42.5%)	296(55.8%)	1.00			
	CT+TT	276(57.5%)	234(44.2%)	1.71(1.33-2.20)	<0.001	1.68(1.30-2.15)	<0.001
Recessive	CT+CC	386(80.4%)	477(90.0%)	1.00			
	TT	94(19.6%)	53(10.0%)	2.19(1.52-3.15)	<0.001	2.05(1.42-2.97)	<0.001
Allele	C	590(61.4%)	773(72.9%)	1.00			
	T	370(38.5%)	287(27.1%)	1.69(1.40-2.04)	<0.001		

*Adjust age, smoking, drinking, and BMI.

**Table 3:** Stratified analyses between rs4789936 polymorphism and the risk of breast cancer.

**Variable**	**Case/Control**			**CT vs CC**	***P***	**TT vs CC**	***P***	**TT vs CT+CC**	***P***	**TT+CT vs CC**	***P***
	**CC**	**CT**	**TT**								
Age											
<60	133/188	104/103	67/36	1.43(1.00-2.03)	0.047	2.63(1.66-4.18)	<0.001	2.29(1.47-3.55)	<0.001	1.74(1.27-2.38)	0.001
≥60	71/108	78/78	27/17	1.52(0.99-2.35)	0.058	2.42(1.23-4.75)	0.011	1.98(1.04-3.78)	0.037	1.68(1.12-2.53)	0.013
Smoking											
Yes	27/33	28/29	38/10	1.18(0.57-2.44)	0.655	4.64(1.96-11.00)	<0.001	4.28(1.95-9.40)	<0.001	2.07(1.09-3.94)	0.027
No	177/263	154/152	56/43	1.51(1.12-2.02)	0.006	1.94(1.25-3.01)	0.003	1.63(1.07-2.49)	0.023	1.60(1.22-2.10)	0.001
Drinking											
Yes	48/64	36/39	31/9	1.23(0.68-2.22)	0.489	4.59(2.00-10.55)	<0.001	1.86(1.10-3.15)	0.021	4.22(1.91-9.36)	<0.001
No	156/232	146/142	63/44	1.53(1.12-2.08)	0.007	2.13(1.38-3.29)	0.001	1.77(1.17-2.68)	0.007	1.67(1.62-2.22)	<0.001
BMI											
>24	84/126	69/53	43/10	1.95(1.24-3.07)	0.004	6.45(3.07-13.54)	<0.001	1.78(0.99-3.20)	0.054	2.67(1.76-4.04)	<0.001
≤24	120/170	113/128	51/43	1.25(0.89-1.77)	0.203	1.68(1.05-2.68)	0.030	2.50(1.58-3.97)	0.000	1.36(0.99-1.87)	0.058

### Gene-environment interactions and breast cancer risk

Using cross-over analysis, we evaluated the gene-environment interactions (smoking and drinking) and the risk of breast cancer (Table 4). For non-smokers and non-drinkers, the TC genotype was not associated with an increased risk of breast cancer compared to the CC genotype. For smokers, the TC genotype increased the risk of breast cancer (OR=1.51, 95%CI: 1.12-2.02, P=0.006) compared to non-smokers carrying the CC genotype. For drinkers, an elevated risk of breast cancer was found in the TC genotype group compared to non-drinkers carrying the CC genotype (OR=1.53, 95%CI: 1.12-2.08, P=0.007). For non-smokers and non-drinkers, the TT genotype was associated with an increased breast cancer risk compared to the CC genotype, but smoking or drinking alone was not associated with an increased risk of breast cancer. However, drinkers and smokers carrying the TT genotype had a significantly increased risk of breast cancer compared to non-smokers and non-drinkers carrying the CC genotype (OR=5.56, 95%CI: 2.74-11.63, P<0.001; OR=5.12, 95%C: 2.37-11.06, P<0.001). These results suggest that the interactions between the genotype and smoking or drinking are associated with increased breast cancer risk in Southern Chinese women.

**Table 4:** Genetic environment factors 2*4 fork analysis.

**G**	**E**	**Case/control**	**OR (95%CI)**	***P***
TT/CC	Smoking			
+	+	38/10	5.56(2.74-11.63)	<0.001
+	-	56/43	1.94(1.25-3.01)	0.003
-	+	27/33	1.22(0.71-2.09)	0.480
-	-	177/263	1.00	
TC/CC	Smoking			
+	+	28/29	1.51(1.12-2.02)	0.006
+	-	154/152	1.43(0.83-2.49)	0.199
-	+	27/33	1.22(071-2.09)	0.480
-	-	177/263	1.00	
TT/CC	Drinking			
+	+	31/9	5.12(2.37-11.06)	<0.001
+	-	63/44	2.13(1.38-3.29)	0.001
-	+	48/64	1.55(0.99-2.44)	0.055
-	-	156/232	1.00	
TC/CC	Drinking			
+	+	36/39	1.53(1.12-2.08)	0.007
+	-	146/142	1.37(0.84-2.26)	0.209
-	+	48/64	1.55(0.99-2.44)	0.055
-	-	156/232	1.00	

### Association between rs4789936 polymorphism and clinical parameters of breast cancer patients

We further explored the association between clinical parameters and rs4789936 polymorphism among breast cancer patients (Table 5). Compared to CC genotype, the TT and CT genotypes were associated with lymph node metastases (TT: OR=2.54, 95%CI: 1.54-4.18, P<0.001; CT: OR=2.10, 95%CI: 1.40-3.15, P<0.001), advanced stage (TT: OR=1.91, 95%CI: 1.17-3.14, P=0.010; CT: OR=1.80, 95%CI: 1.20-2.70, P=0.005), and PR positive status (TT: OR=2.02, 95%CI: 1.23-3.32, P=0.005). The T genotype frequency was not related to tumor size, ER, Her-2, or treatment.

**Table 5:** Association between rs4789936 polymorphism and clinical parameters in patients with breast cancer.

**Parameters**	**CC**	**CT**	***P***	**TT**	***P***	**CT/TT**	***P***
Tumor size							
≥2cm vs<2cm	107/97	92/90		51/43		143/133	
OR (95%CI)	1.00	0.93(0.62-1.38)	0.709	1.08(0.66-1.76)	0.772	0.98(0.68-1.40)	0.890
Lymph node metastases							
Yes vs No	75/129	100/82		56/38		156/120	
OR (95%CI)	1.00	2.10(1.40-3.15)	<0.001	2.54(1.54-4.18)	<0.001	2.24(1.54-3.24)	<0.001
Stage							
III+IV/I+II	76/128	94/88		50/44			
OR (95%CI)	1.00	1.80(1.20-2.70)	0.005	1.91(1.17-3.14)	0.010	1.84(1.27-2.66)	0.001
ER							
Positive vs Negative	92/112	75/107		38/56			
OR (95%CI)	1.00	0.85(0.57-1.28)	0.441	0.83(0.50-1.36)	0.450	0.84(0.59-1.22)	0.363
PR							
Positive vs Negative	86/118	91/91		56/38			
OR (95%CI)	1.00	1.37(0.92-2.05)	0.123	2.02(1.23-3.32)	0.005	1.56(1.08-2.52)	0.016
Her-2							
Positive vs Negative	138/66	132/50		62/32			
OR (95%CI)	1.00	1.26(0.82-1.95)	0.297	0.93(0.55-1.56)	0.773	1.13(0.77-1.67)	0.535
Treatment							
Radiation vs Chemoradiotherapy	74/130	65/117		27/67			
OR (95%CI)	1.00	1.03(0.68-1.55)	0.909	1.41(0.83-2.40)	0.202	1.14(0.78-1.66)	0.503

ER, estrogen receptor; PR, progesterone receptor

### TIMP-2 gene rs4789936 polymorphism and prognosis of breast cancer patients

**Figure 1:**
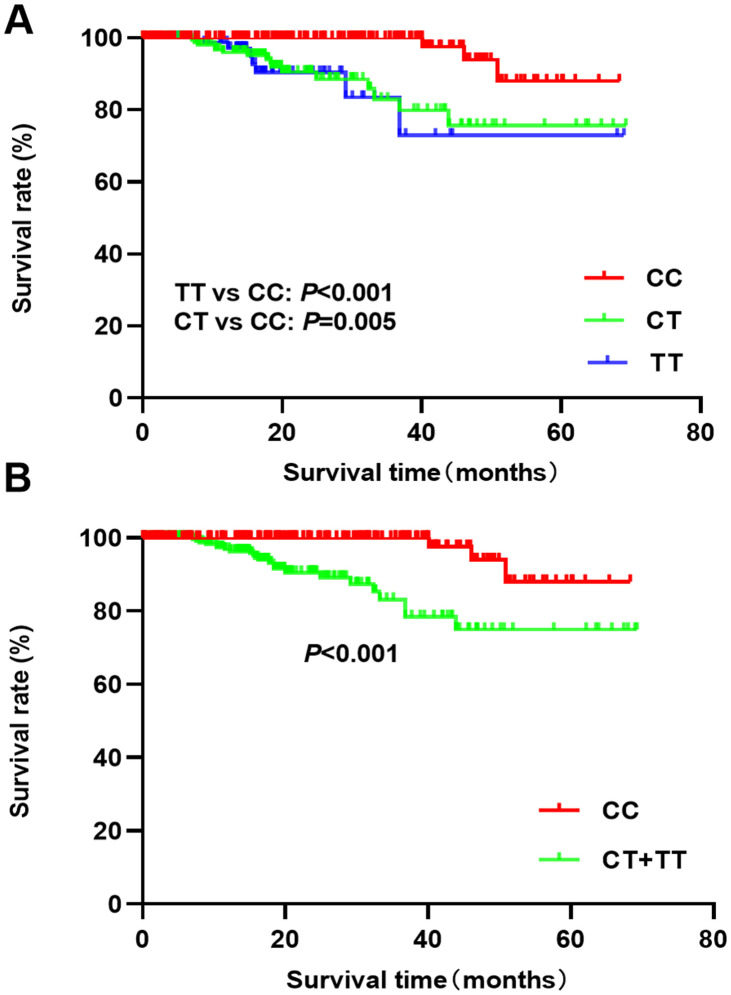
****Kaplan-Meier analysis of breast cancer prognosis and rs4789936 polymorphism ((**A**) TT vs CT vs CC; (**B**) CT+TT vs CC).

Follow-up analysis of breast cancer patients showed a median survival time of 17.6 months. The Kaplan-Meier analysis indicated that the TT/CT genotypes had a poor survival prognosis compared to the CC genotype (CT vs CC: P=0.005; TT vs CC: P<0.001, Figure 1A). Patients with CT and TT genotypes also had poor survival rates (P<0.001, Figure 1B) compared to CC patients. The univariate cox regression indicated that CT and TT genotypes were associated with a poor overall survival prognosis (HR=5.97, 95%CI: 1.99-17.95, P=0.001; HR=5.28, 95%CI: 1.39-20.03, P=0.014). After adjusting the clinical parameters, the CT and TT genotypes (HR=4.65, 95%CI: 1.26-17.18, P=0.021; HR=6.62, 95%CI: 1.51-29.11, P=0.012) were still associated with poor prognosis of breast cancer patients (Table 6). Advanced stage, lymph node metastases, and Her-2 positive status were also predictors of a poor prognosis.

**Table 6:** Prognostic factors in cox regression for breast cancer.

**Factors**	**Univariate analysis**		**Multivariate analysis**	
	**HR (95%CI)**	***P***	**HR (95%CI)**	***P***
Age,year				
≥60 vs <60	1.43(0.64-3.19)	0.386	1.24(0.54-2.83)	0.617
Smoking				
Yes vs No	0.85(0.29-2.49)	0.770	0.76(0.26-2.24)	0.613
Drinking				
Yes vs No	0.36(0.11-1.19)	0.094	0.55(0.15-2.00)	0.365
BMI				
>24 vs ≤24	1.06(0.48-2.36)	0.890	0.87(0.38-2.00)	0.747
Tumor size				
≥2cm vs <2cm	0.82(0.37-1.81)	0.617	0.46(0.20-1.09)	0.077
Lymph node metastases				
Yes vs No	2.35(1.05-5.28)	0.038	2.85(1.03-7.83)	0.043
Stage				
III/IV vs I/II	3.81(1.52-9.51)	0.004	1.91(1.67-4.76)	0.016
ER				
Positive vs negative	0.76(0.33-1.77)	0.524	0.61(0.24-1.58)	0.306
PR				
Positive vs negative	1.40(0.64-3.09)	0.403	0.62(0.26-1.47)	0.273
Her-2				
Positive vs negative	3.79(1.14-12.63)	0.030	2.37(1.73-7.56)	0.041
Treatment				
Radiation vs chemoradiotherapy	2.27(1.05-4.92)	0.037	1.86(0.81-4.29)	0.146
rs4789936				
CT vs CC	5.97(1.99-17.95)	0.001	4.65(1.26-17.18)	0.021
TT vs CC	5.28(1.39-20.03)	0.014	6.62(1.51-29.11)	0.012

## DISCUSSION

The main findings of this study are the following: (1) The TIMP-2 gene rs4789936 polymorphism is associated with an increased risk of breast cancer in Southern Chinese women; (2) There are interactions between SNP rs4789936 polymorphism, smoking, drinking, and breast cancer risk; (3) The rs4789936 TT and CT genotypes are more frequent in breast cancer patients with lymph node metastases, advanced stage, and PR positive status; (4) The rs4789936 TT and CT genotypes are associated with a poor prognosis in breast cancer patients.

Tissue inhibitors of metalloproteinases (TIMPs) are natural inhibitors of MMPs that are overexpressed in many tumors and stromal tissues [23]. The TIMPs have two functional domains: the N-terminal region containing the MMP binding site, and the C-terminal region [24]. TIMP-2 inhibits the MMP-mediated proteolysis, and TIMP-2 decreased expression has been associated with cancer progression. TIMP-2 forms a stable complex with MMP-1 precursor to prevent MMP-1 activation. In addition, TIMP-2 can bind to MMP and directly inhibit its catalytic activity [25]. A previous study has indicated that the dynamic balance between TIMP-2 and MMP-2 can affect tumor invasion and metastasis [26]. A decreased expression of TIMP in tumor tissues results in increased activity of MMP and its increased degradation of the extracellular matrix, thus promoting tumor cell invasion and metastasis [27, 28].

The rs4789936 polymorphism is the transition from C to T that causes a synonymous amino acid change, which can result in dysregulated transcription of the TIMP-2 gene. TIMP-2 gene mutations have been associated with many diseases, including acne vulgaris [29], intracerebral hemorrhage [30], and cancer [31]. A previous study has indicated that TIMP-2 gene rs817990 and rs2277698 polymorphisms affect the risk of primary ovarian insufficiency in women [32]. In addition, two SNPs (rs7501477 and rs8136830) have been associated with an increased risk of breast cancer [33].

A recent case-control study to assess the association between TIMP-2 polymorphism and breast cancer in Northern Chinese population has suggested that the rs2277698 gene polymorphism is associated with a decreased risk of breast cancer [22]. This result is different from our study. Our results show that the T allele of rs4789936 is a risk factor for breast cancer in Southern Chinese women. There are several factors that may explain these differences. First, the eating habits are different between the two populations. In Northern China, people eat cooked wheat food, while in Southern China people prefer rice. Second, the environmental factors are different. It is wet in the South, while the North is dry. Third, there might be a clinical heterogeneity of breast cancer patients from different areas. Finally, some differences in sample size may be a potential factor.

Our cross-over analysis has demonstrated that compared to non-smokers and non-drinkers, women with TT/CT genotype, and drinking or smoking, have a significantly elevated risk for breast cancer, indicating a gene-environment interaction. In addition, we have found that rs4789936 polymorphism is associated with an advanced stage, lymph node metastases, and a PR positive status, indicating that the rs4789936 polymorphism may affect the prognosis of breast cancer patients. A previous study has also suggested that TIMP-2 gene rsrs8136803 TT genotype is associated with a poor disease-free survival [33]. Our data show that the rs4789936 TT/CT genotype is associated with a poor overall survival prognosis compared to the CC genotype, and suggest that the TT/CT genotype might serve as an independent prognosis factor of breast cancer patients. The GTEx data indicate that the rs4789936 TT genotype is quite common in blood samples, and has higher expression levels than the CC genotype. These findings suggest that TIMP-2 regulates breast cancer progression, and that TIMP-2 mutation might result in a poor prognosis in breast cancer patients.

Some of the limitations of this study include the fact that the selected population was recruited from a hospital, and a selection bias may thus exist. Although we identified an association between TIMP-2 gene polymorphism and breast cancer, the potential molecular mechanisms are unclear. There are chances that this SNP has an impact on another gene in which it resides, CEP295NL, because it is position within its 5'UT (HapMap data). Or, probably the truth is that this SNP is in large linkage disequilibrium with a nearby, functional SNP, and further research are required. Besides, since this study only explored gene-environment interactions, future studies should analyze gene-gene interactions that might affect the susceptibility to breast cancer.

In conclusion, our data demonstrate that the TIMP-2 gene rs4789936 polymorphism is associated with an elevated risk of breast cancer in Southern Chinese women, and indicate that it might serve as an independent prognosis factor for breast cancer patients.

## MATERIALS AND METHODS

### Patient population

The study consisted of a case-control population and a follow-up population of breast cancer patients. For case-control population, we enrolled 480 breast cancer patients and 530 healthy controls from the Department of Women’s Cancer, Hunan Cancer Hospital (The Affiliated Cancer Hospital of Xiangya School of Medicine, Central South University). Criteria for enrollment were the following: All cases were newly diagnosed by pathology diagnosis; they were Han ethnic, and aged from 25 to 70 years old. Cases with other types of tumors, cardiovascular diseases, severe infectious disease, and immune disease were excluded. The control population was recruited from the physical examination center of the hospital during the same period. The follow-up population was from the case group.

General data of all human subjects were collected including age, height, and weight for calculating the body mass index (BMI), and the history of smoking and drinking. For the case group, we also collected the tumor size, lymph node metastases, stage, the status of estrogen receptor (ER), progesterone receptor (PR), Her-2, and treatment. A telephone follow-up was performed, and the primary outcome was overall survival (defined as the time from surgery to death). This study was approved by the ethics committee of Hunan Cancer Hospital. Written informed consent was obtained from all study subjects.

### Blood sampling and genotyping

rs4789936 with minor allele frequency> 5% in East Asian population was selected for this study. 5 ml of fasting peripheral blood was collected from each participant, and was stored in anti-coagulative tubes with EDTA-disodium salt. The genomic DNA was extracted by TaKaRa Genome DNA Extraction Kit (Dalian Biological Engineering CO., LTD, China), and stored at -20°C.

The single nucleotide polymorphism (SNP) was genotyped by polymerase chain reaction restriction fragment length polymorphism (PCR-RFLP) method. The primer sequences of TIMP-2 gene rs4789936 were designed by Primer Premier 5.0, and synthesized in Shanghai Sangon biotech Co., Ltd (ACGTTGGATGGCGTC, TCACTACCTACAAAG). The PCR reaction was performed in a total volume of 25 μl, including 5.0 μl of DNA template, 2.5μl of 10×Buffer, 2.0μl of MgC12, 0.5μl of dNTPs, each 0.5μl of forward and reverse primers, 0.2μl of Taq enzyme, and 13.8 μl of ddH_2_O. The amplification conditions of PCR were as follows, starting with pre-denaturation at 94°C for 2 min; followed by 36 cycles of 94°C degeneration for 20s, 53°C annealing for 30s, 72°C extension for 30s; and finally, 72°C extension for 10 min. Purity of the PCR products was examined by 2% agarose gel electrophoresis. The PCR products of rs4789936 were digested, and the digested products were analyzed by 3% agarose gel electrophoresis and visualized by UV light. Random samples of the PCR products were selected for direct sequencing to verify the accuracy and reliability of the genotyping results.

### Statistical analysis

SPSS 20.0 statistical package was used to perform the statistical analyses. The quantitative variables were expressed using mean±standard deviation, and t test was used for the comparison between case group and control group. The genotype frequency of cases and controls was compared using the Chi-square test. Hardy-Weinberg equilibrium test was performed in the control group. For rs4789936 polymorphism, we used five models for compassion: homozygote model (TT vs CC), heterozygote model (CT vs CC), dominant model (CT+TT vs CC), recessive model (TT vs CT+CC), and allele model (T vs C). Univariate and multivariate (adjusting for age, BMI, smoking, and drinking) logistic regression analyses were used to calculate the odds ratios (ORs) of breast cancer, and the 95% confidence intervals (CIs). Stratified analysis was performed according to age (≥60 vs <60), smoking (Yes vs No), drinking (Yes vs No), and BMI (>24 vs ≤24). The cross-over analysis was used to estimate the gene-environment interactions (gene-smoking, gene-drinking). We also explored the relationship between clinical parameters and genotype distribution (tumor size, lymph node metastases, stage, ER, PR, Her-2 and treatment). The Kaplan-Meier analysis was used to compare overall survival (OS) among different genotypes. Univariate and multivariate (age, smoking, drinking, tumor size, lymph node metastases, stage, ER, PR, Her-2 and treatment) cox regression analyses were used to determine the relationship between genotype and breast cancer prognosis. Hazard ratio (HR) and 95% CI were also calculated. *P*<0.05 was considered significant.
